# The body as constitutive element phenomenology and psychoanalysis on our view of ourselves and others

**DOI:** 10.1186/s40504-017-0051-0

**Published:** 2017-04-26

**Authors:** Paulina Monjaraz Fuentes, María del Carmen Rojas Hernández, Stefano Santasilia, Fernanda Monjaraz Fuentes

**Affiliations:** 1 Faculty of Social Sciences and Humanities, Universidad Autónoma de San Luis Potosí, San Luis Potosí, México; 2 Faculty of Psychology, Universidad Autónoma de San Luis Potosí, San Luis Potosí, México; 3 División de Neurociencias, Departamento de Neuropatología Molecular, Instituto de Fisiología Celular, Universidad Nacional Autónoma de México, Ciudad de México, México

**Keywords:** Body, Biology, Medicine, Phenomenology and psychoanalysis

## Abstract

The aim of this manuscript is to highlight that from the phenomenology and psychoanalysis point of view, the meaning of the notion of the body is different from the medical *biologicist* discourse. In psychoanalysis, the body is an erogenized body. It is constituted as an object for another self. Similarly, in phenomenology, the body is an own body in first instance. It is the body of a self, rather than a living body and a material body. Both positions enable us to understand how this conceptualization of the body is essential in any human field. Especially in the clinic, the position of the subject before the other will lead to a specific form of intervention. From this understanding of the human body, both phenomenology and psychoanalysis confirm that the *biologicist* understanding of the body, presumed by all psychological and medical practices, is insufficient.

## Introduction

The mind-body relation (psyche-soma) has always been the central topic of philosophical anthropology and psychology. This ancestral and current debate is the irremediable framework when speaking of the body. Succinctly, the central question is whether or not the mental/psychic operations, acts or states (seeing, imagining, feeling, thinking), are different from physical processes (particularly neural processes), and the relation between them. If there is no source of motion other than the physical, then the body can be understood in its entirety only by the study of the body as a biological organism. Such a stance has prevailed in the understanding of the body within medicine, largely because of the philosophical postures that have been adopted in the mind-brain or mind-body problem. In general, without pretending to include each philosophical position with their nuances, all these postures reduce activity- or the cause of activity- to a material cause.

The philosophical positions regarding the question of psyche-body relations through history can be broadly divided into:

Dualism claims a real distinction between soul and body. Likewise, the Psychophysical Parallelism usually recognizes some distinction between the mental and the physical but disregards or does not admit their mutual interaction (postures of Leibniz and Spinoza). On the other hand, the Spiritualist Monism denies the notion of body as something really different from the spirit or knowledge (idealism of Berkeley). Moreover, the Psychological Behaviorism attempts to solve certain inner attitudes within the neurophysiological stimulus-response scheme, subjecting these actions to the rigor of the natural sciences (Skinner), and the Philosophical Behaviorism explains internal processes through external or public behavior (Ryle). Furthermore, the Neurologist Monism, or identity theory, reduces the psychic act and its intentional contents to neuronal activity, so it ends up being called physicalism (Paul and Patricia Churchlan). Then, Emergentism comes about as an opponent to neuronal reductionism, although both positions emerge from a material organization principle (Searle, Bunge). Successively, Computational Functionalism arises as an explanation for mental acts and states contrary to behaviorism and neurologism, arguing that mental operations could be computational functions capable of being realized in multiple ways in various material bases (Putnam-at the beginning). The result is a new extreme dualism, since the mental functions could happen independently of the material structure. Consequently, body and mind could exist totally separated. (Sanguineti, 2008).

In accordance with Sanguineti, all the positions that reduce mental or psychic activity to physical causes, where the biologicist medical position lies, are challenged to sustain a strict materialist theory coherently in the following three aspects: a) The *I*, the s*ubjetivity* (or the conscience, or the *qualia problem*) is eventually dissolved in neural and computational reductionism, although artificial constructions may remain; b) *Intentionality*, a relationship that makes sense only if it recognizes the reality of knowledge; c) *Rationality*, taken as a non-physical cause or physico-nomological explanation of intentional human behavior: “to act for a reason” and not simply due to some third-person neural determinism. If rationality and the *I* are admitted, freedom is implicitly recognized. In neurologism or computationalism, freedom is dissolved or reduced to simple undetermined behavior “(Sanguineti, 2008).

Given the prevalence of such reductionism, mainly in the “cure” of mental illness proposed in the *Diagnostic and Statistical Manual of Mental Disorders*, Fifth Edition (DSM-5), the aim of this work is to show how from the phenomenological and psychoanalytical posture, we cannot speak of the human body only from an organic-biological understanding. The latter understanding of the body reduces its object of study to the material by admitting as valid only the experimental scientific method. In this framework, the body can only be explained under the epistemological assumptions of the experimental sciences (natural sciences). Thus, it reduces the body to a material (biological) reality, and it denies the existence or rational validity to everything that does not fall under such epistemological consideration.

These conceptual challenges are not exclusive to theoretical philosophy, “practicing clinicians and primary care physicians are confronted with the full and uncategorized complexity of human function and dysfunction on a daily basis”. However, scientific (reductionist) medicine prevails in today’s medical practice, which attempts to reduce the personal “psychosocial” to molecular and cellular mechanisms or programs in the brain. However, the ontological mind-body dualism persists in medical practice, since these biological mechanisms do not disappear the subjective personal experience (Miresco and Kirmayer 2006).

As science is aligned with the philosophical reductionist posture, and medicine aligned to science, scientist medicine explanation, which is currently dominant, lays in the machine metaphor, which describes all living entities as machines. “The patient is seen as “nothing but” parts in interaction and should be understood, studied, and treated by focusing on those parts. The patient thus becomes a *passive* and *static* thing rather than an *active* and *dynamic* process” (Bickhard 2011).

There is evidence of diseases of the psyche resulting in diseases of the body, and vice versa, diseases of the body resulting in diseases of the psyche, and also diseases of the psyche that cannot be allocated in the body (or brain). Prior to the disease, there is an *I* experiencing the disease. This shows, again, that the body cannot be understood without the psyche, the physical and non-physical coexist and each cannot be explained without the other. We can understand a physical mechanism but that does not lead us to understand how the subject experiences it. This cannot be neglected by sciences dealing with the human being (i.e., medicine), since experience, human subjectivity, is not isolated in disease, in neither disease of the body or the psyche.

The relation of the human being to the World is a very important part of experience, thus science has focused on the processes of physical sensation. We know physical sensations are integrated in the brain, thus we look for the manifestation of the mind or consciousness within it. Cognitive neurosciences research misinterpret this and assume that the mind is instantiated in the brain, and aims to explain how mental phenomena (perception, memory, attention, learning, etc.) are generated in the brain. Thus, Cognitive neurosciences presumptions are that the brain, by means of neural process, generates the mind. The basic working assumption of cognitive neurosciences is that the mental events are nothing more that a special kind of physical events (Repovs 2004). However, the individual, its subjectivity, co-founds the structures, functions, neural processes, social and cultural contexts in which the mind is placed. Neuroscience neglects this, but is crucial for a wholesome understanding of the human being, as constituted by its inseparable body-psyche. Neurosciences, usually equating consciousness to awareness, is incapable of explaining the qualitative experience that accompanies neural processes. However, if cognitive neuroscientists escape reductionism and acknowledge the diverse philosophical postures, then they could have a better approach to the study of consciousness by reformulating their experimental questions and hypotheses, even if their experimental procedures are limited to the physical.

The complex body-mind relationship is addressed early in psychoanalysis, specifically in the text *“Psychological Treatment (soul treatment)”* written by Freud in 1890. In this manuscript, he alludes to the difficulties that physicians have had in scrutinizing the mutual influences exerted between the body and the psyche. The text starts saying “*Psique* is a Greek word translated to German as “*Seele*” (*soul*), so it can be argued that psychic treatment is the same as soul treatment” (Freud 1890, p. 115). Freud was referring directly to a confusion that usually occurs when understanding psychic treatment as soul treatment, separating the *psyche* from the body. This confusion not only occurred at that time, but also prevails nowadays.

To face this confusion, Freud clarifies that the “psychic treatment” rather means treatment *from the soul*, either from psychic or bodily perturbations, with resources that influence primarily and immediately the psychic of the man” (Freud 1890, p. 115). Therefore, Freud claims that *the word* is the only resource to produce a healing psychic influence, for the discomforts of the soul and consequently also the body. At the same time, he criticized medicine for its great progress as a science in the XIX century, under the happy influence of natural sciences, while disengaging from the psychic processes of the man.

Freud acknowledged the success of medicine and the natural sciences regarding the progress in understanding the anatomy and physiology of the human body, the role of microorganisms in the origin of many diseases, the signs of many morbid processes, and many others. However, he remarks, “All these progresses and discoveries are concerned to the human body. Hence, as a result of an incorrect (but understandable) judgment orientation, physicians shifted their interest to the body and left philosophers, whom they despised, to deal with the psyche” (Freud 1890, p. 116).

From a diverse perspective, Husserl addressed the same issue and analyzes the situation of science in the late XIX century and early XX century in his work “The crisis of European sciences and the transcendental phenomenology” (1934–1937). He criticized the sciences that claimed to be “natural” and aspire for “purity” or “objectivity” through the experimental scientific method, in order to separate science from all mixture of subjectivity. He remarked that they ignored an essential aspect of science, which is that science itself is a creation or production of human subjectivity. Subsequently, Husserl added that such an “aspiration” would denote that one who thinks is not a subject or a consciousness, which is not only false but also absurd. Regarding this crisis, Freud noted that modern medicine studied in depth the nexus between the physical and the psychic but “in no case it ceased to present the psyche as commanded by, and dependent of, the corporal. […] They seemed to fear [the physicians] that if they granted certain autonomy to the psychic life, they would no longer be standing on the safe ground of science” (Freud 1890, p. 116). Nevertheless, in the same document Freud relates that despite this trend followed by the physicians, a parenthesis began to open due to evidence of patients showing discomforts, to which science could not allocate their cause in the body. Consequently, the medical field labeled this patients as nervous or neurotic, and accepted that the origin of their diseases came from “an altered influx of their psychic lives over their bodies” (Freud 1890/1891. p.118).

## Experience and body

In psychoanalysis, the body is an erogenized body, implying the body is a “signified” body. The “erogenized body” refers to a “ body of desire”, which functions as a symbol endued with significance. Freud modified the notion of the body that prevailed during the XIX century through his incipient clinical practice, which shifted the focus of attention from the symptom to *the listening* (what the patient *said*). This practice opened a new way of understanding the body, thus becoming the *said body* inscribed and expressed by the language.

In the development of the psychoanalytic theory there is a constant allusion to the body. The notion of the unconscious, the cornerstone of psychoanalytic theory, cannot be understood in any way without considering its relation to the body. Therefore, it is important to emphasize that the body referred to in psychoanalysis is not the organism treated in the medical discourse. Moreover, psychoanalysis does not contribute to the knowledge of the biological body nor does it intend to do so. In this line, some of the Lacanian contributions propose to understand the body as something more than the living as biological. Lacan proposes that the body is not the primary thing; the subject is not born with it. So understanding the body as a living organism is not enough. Rather, to have a body requires that living organism, but also requires an image of itself. The subject apprehends its own image as a unit like a specular effect that produces the look of another. However, the organism is discordant with itself and at first is perceived by the child as a series of fragmented sensations as long as they are not integrated by an image. The other provides this image, resulting in an imaginary identification. Lacan (1970) relates this identification with the instauration of the *I*, the imaginary *I*. In “Radiophonie”, he approaches the body from the symbolic, that is, the incorporeal body that he considers a “gift” of language.

The subject is addressed before having a body and is also present when his body is damaged, even when his body dies, as anthropology confirms with the study of graves. “The true body, the first body, is language, which Lacan later called” “the symbolic body”.

The symbolic body is indeed a body, because we can consider it as a system of internal relations. The language is body, and in addition it is a body that gives body” (Garrido 2010). Hence, the organism is not in the language and Lacan considers it as the Real. The organism is the irreducible to language and to knowledge, that which is present but not known. Consequently, Lacan referred to the three registers: Real, Symbolic and Imaginary to address the body as a ensemble of identifications. Hence, the great technological advances are not sufficient when applied to assist the diseases of the body. Neither the technology nor other devices of assistance are sufficient when used to attend the organism by themselves. Therefore, the medical sciences need to incorporate other discourses based on an understanding of the body that includes subjectivity, instead of focusing only in the organic of the body.

Since its origin, psychoanalysis has dealt with a body that has been excluded from the field of work and research of medicine. The medical-scientific discourse leaves out the constitutive subjectivity of the human being. Thus, psychoanalysis emerges precisely to deal with what science discards regarding the knowledge and wisdom about the body, i.e., passions, emotions, affections, and all of the expressions of subjectivity closely related to the body. Almost a century after the emergence of psychoanalysis, Lacan (1971–1972) stated that “the analytical discourse is not about a scientific discourse, but a discourse for which science provides the material” (p.73), because what is not within the interest of science about the body is just what constitutes the study object of psychoanalysis.

In reference to the above mentioned, psychoanalysis, concretely the Freudian theory, departs from the dualist body-mind concept and introduces a field for which the body and affections cannot be considered separately but in close conformation. Accordingly, the concept of *drive* becomes part of the theoretical backbone of psychoanalysis. The *drive* is a “frontier conception between the psychic and the somatic, like a psychic representative {Repräsentant} of the internal stimuli coming from the body and reaching the soul, like a measure of the labor demand imposed to the psychic as a consequence of its interconnection with the bodily” (Freud 1915, p.117).

Simultaneously, Husserl proposed a phenomenology that establishes the epistemological basis to distinguish between Subject and World. Subject is precisely a consciousness, and everything that is not consciousness is World. In phenomenology, the intentionality of the consciousness^1^ implies that it always has to be owned; in other words, the consciousness always has an object. Husserl remarks how the “things there” respond to rigid and inseparable causal laws between them.

“The thing is constant in that it comports itself in such and such a way under the circumstances which pertain to it: *reality* (or, what is here the same, *substantiality*) and *causality belong together inseparably.* Real properties are *eo ipso* causal ones. To know a thing therefore means to know from experience how it behaves under pressure and impact, in being bent and being broken, when heated and when cooled, etc., i.e., to know its behavior in the nexus of its causalities: which states does the thing actually attain and how does it remain the same throughout these states” (Ideas II, §15, [45]).

Certainly, the human body has a physical-biological dimension but it is not ruled solely by this movement principles. Husserl proposes phenomenology as the knowledge of “essence”, which explains what things are and not just throws “data” on them. Hence, phenomenology is radically distanced from all forms of empirism and positivism. Such an epistemological path affirms that the only way for humans to approach “reality” or things “lies on the consciousness”, since that who knows is a “subjectivity”, i.e., a consciousness. This implies that everything is presented to the human being as a phenomenon. To avoid falling into an empiricist posture, we must explain that the phenomenon is not “objective data” but rather something given through experience. Phenomena appear to the senses, and may or may not correspond to what actually exists in the natural world, so a possible correspondence is not guaranteed. Consequently, the phenomenon is not identical to the real object that is manifested, but is simply the manifestation itself. Hence, phenomenology establishes epistemologically, through an essential analysis of the consciousness, the different ways “things” are presented to the consciousness.^2^

Edith Stein, disciple and temporal assistant of Husserl (father of Phenomenology), analyses the constitution of the psychophysical individual in her doctoral thesis “The problem of empathy”. She explains that, in phenomenology, the own body is the first thing presented to experience through *presentification,* because the “free movement” of a human body is evident in the presence of a human. In other words, the human body is not subjected only to the physical laws, but moves from itself. Therefore, the first thing presented to experience is the body of a subject or the body of a consciousness, either its own or the other’s. Edith Stein synthetically states: “the own of the human body as an organism is: information of the matter by the internal *vital form*, the joint action of the necessary structural materials, the gathering of parts in the all, the generation of other individuals of the same species. This whole process, which we call life (*Leben*), is activity (*Tätigsein*), and activity is movement (*Bewegung*) ” (Stein 1932, p.39). 3 The living body has sensitivity so –through this– it is presented to us as jointed to the subject, since one feels, and this corporality is linked to an individual consciousness. The inseparability between body and subject is not only spatial, since sensitivity is a quality occupying an entire extension and not located only in a place of the body. This sentience occupying the entire extension of the body can be named “impressionability” or “sensitivity” (*Empfindsamkeit*) (Stein 1920/21, §322).

The body is linked to a consciousness because is owned, it feels everything and so does the subject. Therefore, sensitivity can be distinguished from mere sensation. The first thing presented to experience is the body as subject, or, the body or a consciousness of our own or other’s. It is necessary to clarify that consciousness in phenomenology is not ‘to realize something’, but the structure allowing the constitution of an *I*, i.e., of a ‘self’. The self will be the continuous flow of the subject’s experiences, therefore, consciousness is not a ‘full’ structure, but rather a structure that will be shaped according to experiences and how it receives and shows all that is presented to consciousness, or what is not happening out there (objective), but that ‘happens to it’.

As explained before, the phenomenological approach allows us to analyze the body not only as a ‘thing there’, because although it certainly is a physical thing with biological characteristics, it is not presented at first instance as a thing out of me. The body is not a ‘being in there’, an object at hand, precisely because the hand is my body. In this way, the body is not presented to the consciousness as ‘something’ de-subjectivized, as a material being in there, outside of me -or- of the consciousness, but it is first and foremost an own body. Therefore, Freud, in phenomenology, clarifies why the body cannot be separated from the psyche.

## Spatiality and body

Freud (1923), in his manuscript “The Ego and the Id” establishes a guiding principle to understand the psychic apparatus stating that “a person’s own body, and above all its surface, is a place from which both external and internal perceptions may arise. It is seen like any other object, but to the touch it yields two kinds of sensations, one of which may be equivalent to an internal perception.” (Freud 1923, p. 27). Hence, the internal perception is an immediate reference and constitutes the basis for all perceptions and representations.

Phenomenology explains how the internal perception is formed and why is internal and not external. The own body is not constituted as an object of external perception, i.e., as a physical (natural) body, because the subject perceives his body as belonging to him. In contrast, for the subject, the external bodies are always there or absent. The body of a subject is always at the same reaching distance to grasp it,^4^ meaning that it is always here whether one sees it, touches it, hears it, or not. One’s body is always ineluctably present as a full “own corporality”. To be linked or to belong to oneself can never be constituted through an external perception, but always through an internal perception (Cfr. Stein 1917, pag. 122–123).^5^ Therefore, in phenomenology, the human corporality is understood mainly as the body of a consciousness. The “own body” is an object that is given to me as a sequence of variable appearances. However, if the giving of my body is compared to the giving of the other physical bodies, then my body is given to my sensibility within very narrow limits.

The limits of the sensorial perception of the own body avert a unitary perception, and thus avoid the presence of the body from being like a shapeless mass or an undifferentiated presence. On the contrary, the body is constituted according to the form of sensations, which implies a variable distance from the self. This variable distance between some parts and the body refers to a zero point of corporeality with respect to the self. One perceives the parts of the body to be further or closer from this zero point, but regardless of the distance the parts belong to one, i.e., they are always internal (Cfr. Stein 1917, pag. 122–123).

Nevertheless, it is important to note that the body is not the same as the self, because my body cannot be identified with the *I.* 6 However, the body and the self are linked to the extent that the body is located a zero distance from the *I*. From this point, the body parts, more or less distanced from the *I* and simultaneously integrated in what the *I* is, are considered as part of *one’s own body* (Stein 1917, pag. 123–124). Space is mentioned as a reference of the body parts towards the *I*, because the subject is perceived as a whole unit. Therefore, the outer spatiality related to one’s body is oriented towards one’s corporality. Similarly, Freud, in his book “The ego and the Id”, considers that “psycho-physiology has fully discussed the manner in which a person’s own body attains its special position among other objects in the world of perception […] The ego is first and foremost a bodily ego; it is not merely a surface entity, but is itself the projection of a surface (Freud 1923, p. 27). Later in 1926, he adds a footnote to the same document and admits that the *I* should be considered as a psychic projection of the surface of the body, as well as the representation of the surface of the psychic apparatus (Freud 1923, p. 28, footnote).

## The own body and the foreign body

In phenomenology, the relationship between internal and external perceptions denotes the intentional character of the consciousness, because the consciousness is always owned. Also, this owner cannot be just oneself, the other (the external) is required. Hence, the I is always present in the consciousness. Nevertheless, the i is not constituted as I without the other or the external. Therefore, the external perception of “other bodies” is required, i.e., the foreign and what the no corporeal of the I, for the identity of the I to be constituted. Consequently, the identity -of the own- would not make sense if the -not own- were not presented. Hence, identity needs alterity.

In the dynamics between identity and alterity lies the essential relevance of the internal and external perception. So that, in this dual mode, internal and external, the own body comes alive as the same body (Stein 1917, pag.125), and the foreign body as the not-own-body. Husserl (Ideas I, §.67) emphasizes the importance of not confusing sensation and perception, because sensation is blind and deaf. In other words, sensation only is meaningful if perceived, because it requires that close relationship between body and consciousness to be constituted in the experience of the I.

The language between sensation and perception is particularly narrow, because the own body is given as a sentient and the sensations are the data from the own body. Sensations are given in an absolute way, since they are always localized. The sensation is always in a certain place of my body, but always distant from the self. It may happen close to the I, but never in I. All this places where sensations are manifested are gathered in a unit that is the own body (Cfr. Stein 1917, p. 129). Likewise, as mentioned before, Freud stated, “a person’s own body, and above all its surface, is a place from which both external and internal perceptions may spring. It is seen like any other object, but to the touch it yields two kinds of sensations, one of which may be equivalent to an internal perception.” (1923 b*, pg. 27). Certainly, the own body is perceived through the external sensibility. However, this perception is not a simple sensation but a perception constituted from the I as a perception of itself. Simultaneously, the laws proper to physical things also constitute perception as they are presented to consciousness. Therefore, my own body is constituted in two ways: as a sentient own body (perceived as own body) and as body of the external word (perceived exteriorly). The own body solipstically constituted, seen from the interior - in the versed approach on the “interior”, is manifested as a free mobile organ (or a system of organs) through which the subject experiences the external world. Moreover, as a bearer of sensations, the own body -and the psyche- form a concrete unit, due to the link it has to the rest of the psychic life (Husserl, Ideas II, §. 42. p. 162–163).

Nonetheless, the internal and external are intertwined as they emerge in consciousness, as Husserl remarks.

“Approached from the outside –in the “outer attitude”– it presents itself as a reality sui generis. That is: on the one hand, as a material thing of especial modes of appearance, a thing “inserted” between the rest of the material world and the “subjective” sphere (the subject together with what was just mentioned from within), as a center around which the rest of the spatial world is arranged, and as being in causal relationship with the real external world. On the other hand, the Body appears here at the same time as a “turning point” where the causal relations are transformed into conditional relations between the external world and the Bodily-psychic subject. And in virtue of that, the Body appears as pertaining integrally to this subject and its properties, both the specifically Corporeal and the psychic ones bound up with them. That which is constituted in the outer attitude is there co-present together with what is constituted in the inner attitude” (Ideas II, §.42, p.161–162).

Therefore, the objective world (external) and subjective world are intertwined inseparably as consciousness arises, but this does not mean they cannot be distinguished, let alone identified. However, in the subject’s life, or constitution as a psychophysical individual - the constitution of its identity-, this relationship is intentional, of meaning or signification. Thus, the human body, either the own body or the body of other consciousness, cannot be known outside subjectivity, since it would no longer be a body and would be a “thing there”. We give meaning to the “thing there”, but it is not an essential part of our own significance, or identity.

Husserl’s research, evidently oriented in a transcendental direction, although at first might not seem that way, grants to the corporeal reality a place in the foundation of the same subjectivity, without leaving it at the level of the “sensible underground”. This transcendental orientation, like the thought of Edith Stein, does not affect the whole phenomenological perspective. Therefore, other postures develop in phenomenology, one of them is the posture of Maurice Merleau, which especially addresses the corporality topic, and adopts a particular perspective through a reinterpretation of the Husserlian program.

In the Prologue of “Phenomenology of perception”, Ponty announces: phenomenology is also a philosophy which puts essence back into existence, and does not expect to arrive at an understanding of man and the world from any starting point other than that of their “facticity” (Merleau Ponty 1945, p. vii). According to the philosopher, such a statement has a precise consequence that he explains: “the phenomenological world is not a pure being, but the sense which is revealed where the paths of my various experiences intersect, and also where my own and other people’s intersect and engage each other like gears. It is thus inseparable from subjectivity and intersubjectivity, which find their unity when I either take up my past experiences in those of the present, or other people’s in my own” (Ponty 1945, p. xxii). The French thinker proposes to start the research again in order to work differently from what has be thought so far according to some precise categories. If at the end of the cited Prologue we read an invitation to recover a sense of philosophy that is not the recognition of a previous reality but relearning to see the world,^7^ surely this task is radicalizes in his following work “The visible and the invisible”.

Maurice Ponty considers the body as a fundamental structure, especially chiasmic, that allow us to place ourselves within the, happy term, “flesh” of the world (Merleau Ponty 1964, p.134). This locality reveals the body as the only possibility of subjectivity and communication. In this few statements, in a way, the transcendental Husserlian posture is already broken, because there is no ontological primacy of the spiritual over the material and instead the flesh of the world is the fundamental element. Like Husserl, for Ponty the tactile sensation dominates the panorama, and since all eyes are conditioned by the same body movements, the French author can decisively affirm that the visible and its tactile qualities belong in the same way to the touch: “We must habituate ourselves to think that every visible is cut out in the tangible, every tactile being in some manner promised to visibility, and that there is encroachment, infringement, not only between the touched and the touching, but also between the tangible and the visible, which is encrusted in it, as conversely, the tangible itself is not a nothingness of visibility, is not without visual existence (Merleau Ponty 1964, p.134)”. Consequently, the term of “own body” disappears in Ponty's discourse, since the body presents a reciprocal relationship with the world because is inserted in its own “flesh”. The body-I is already world, however, matter is always an expression of the I in its horizon of meaning.

In Ponty’s phenomenology, the visibility arises from belonging to the same visible, through a locality in which the tangible and the visible are always attuned without being mixed up. One who sees can only possess the visible if the visible possess the one: We understand then why we see the things themselves, in their places, where they are, according to their being which is indeed more than their being-perceived- and why at the same time we are separated from them by all the thickness of the look and of the body; it is that this distance is not the contrary of this proximity, it is deeply consonant with it, it is synonymous with it. It is that the thickness of the flesh between the seer and the thing is constitutive for the thing of its visibility as for the seer of his corporeity; it is not an obstacle between them, it is their means of communication (Merleau Ponty 1964, p.135).

The flesh constitutes the world since the body is the thickness that allows me, as Ponty explains, to go to the heart of things and turn them into flesh, which is what I am: “The body unites us directly with the things through its own ontogenesis, by welding to one another the two outlines of which it is made, its two laps: the sensible mass it is and the mass of the sensible wherein it is born by segregation and upon which, as seer, it remains open” (Merleau Ponty 1964, p.136).

For Ponty, the flesh that underlies the same corporeality is not merely matter but neither is spirit. He clarifies with his own words: “To designate it, we should need the old term “element”, in the sense it was used to speak of water, air, earth, and fire, that is, in the sense of a general thing, midway between the spatio-temporal individual and the idea, a sort of incarnate principle that brings a style of being wherever there is a fragment of being” (Merleau Ponty 1964, p.139). The flesh, as an element, is the same location, the possibility of any fact and experience, in a word, affirms the philosopher, the facticity. Therefore, the flesh is a general element, but also individual, in the structure through which there is always a body of the spirit and the spirit in the body. The flesh is the junction of multiple “entrances” to the world that philosophy has to indicate (Merleau Ponty 1964, pp. 259–260).

The flesh referenced by Ponty is the new chisamic definition that manages to maintain united the own body and the world, still being a possibility of each fact, and therefore also a principle of subjectivity. This definition is still too general, and ends up losing, in some way, the adherence to the individual life of the subject that would support the carnal condition as a corporeity depth. However, in accordance with the analysis presented here, and despite its phenomelogical radicalism, Ponty’s thoughts on corporality support the Husserlian and psychoanalytic intuition concerning the impossibility of treating the human body as mere matter. The need of the French phenomenologist to introduce the term “flesh”, in order to escape from the total “spiritualization” of the body as well as its total “materialization”, admits the hallmark of the phenomenological recognition to a corporality that cannot be reduced, in any way, to mere physiological mechanics.

## Conclusions

As we have shown, both the psychoanalysis and the phenomenology consider that the human body cannot be explained rigorously from the human corporality, leaving behind its subjective dimension, which implies that the body is a constitutive and inseparable part of the conscious subject complex. Thus, the binomial-psyche cannot be separated when addressing the human body thoroughly.

Both psychoanalysis and phenomenology assert that the subject’s body is always an “own body”, since it bears a unique character in spite of the organic similarities among all bodies. The “own being” of the subject cannot be explained from the causality of material things, because they are always external to the subject, they are world and follow a rigid law, they are physical. If the explanation of the subject’s body is limited by considering its movement mode only from the physical causality, then the “free movement” inherent to it, the fact that it is not subjected only to rigid physical laws, would not be explained. Consequently, such an explanation would dismiss the position of the corporality of the subject before the world, and that it is not in the world just as a “thing there”.

The individuality, or rather ipseity, which presumes having an own body, could not be explained from natural sciences, given the dependence of causalities on natural law. If one restricts the understanding of human corporality to its biological-physical dimensions, then one would have to accept that this consideration, by method, has left aside the dimension of the subject, and therefore cannot make any assertion regarding its subjectivity. Thus, in this understanding, the approach to an inert body, or an animal body, or a dead body, would be the same, since it is restricted to not consider the movement of that body, and cannot distinguish the difference between them.

Given the arguments presented in the current manuscript, we need to state that the natural sciences, limited by the experimental scientific method (positivist), can know the human corporality exclusively as a material body, in their causal relations and as physical substances. Consequently, the proper of the human is left out of the scope of natural sciences, thus they are incapable of giving a complete and unified view of the human corporeality. The human body cannot be considered as an organic entity exclusively, because in doing so it leaves out the subjective production that implies, concerns and affects the body, which is equally constitutive of the subject and inherent to the body.

The above mentioned could explain the inefficiency and inefficacy of current medicine to “heal” the body, even more so to explain the psychic sufferings from the biochemistry or neuroscience understanding. The methods proper to the experimental sciences nullify the possibility of approaching the understanding of the mental processes and their therapeutic. These sciences make classifications under statistical arguments intended to establish a synonym between the observable behaviors and their presumed correlative mental processes, as is the case of the DSM-V. Thus, they leave behind the central object of study, that is, the subjectivity that coexists with the human body. This subjectivity that makes the own body - as it has been sustained in this article- cannot be reduced to matter, or factual data. If the human body is not considered as the origin of movement and different from matter and physical principal, then, yet again, we neglect that which distinguishes the world from consciousness, that which gives meaning to everything that surrounds the man and prevents the body to be reduced to a “ being there”, to a mere object, meaningless, to an “object” to be used as something there. Also, it is neglected that which makes the body being owned, an incarnated spirit, a body of a subject that interpellates subjectivity to recognize the other as another subject like the *I*.

However, if cognitive neuroscientists escape reductionism and acknowledge the diverse philosophical postures, then they could have a better approach to the study of consciousness by reformulating their experimental question and hypothesis even if their experimental procedures are limited to the physical.

## Footnotes

1 The conciousness in philosophy is not the “realization of something”, but is the structure that allows the constitution of the *I,* that is, of the “self”. The *I* is the continous flow of the subject’s experience. Therefore, conciousness is not a “full” structure, but rather a structure that will be shaped according to experiences and the way it “receives” and “manifests” everything that is presented to the consciousness, or that not only happens out there (objective), but also “happens to it”.

2 Husserl’s phenomenological philosophy, placed in consciousness, gives rise to important phenomenological developments about the body. One of them is Sartre’s understanding of the body as “the body for me” and “the body for the other.” Also, due to the the phenomenological approach inaugurated by Husserl, Merleau Ponty conceives the body-psyche unit as a *lived body* and the subject as an *incarnate consciousness*.

3 “Körper als Organismus eigen ist: die Formung der Materie durch die lebendige innere Form, die Aneignung der nötigen Aufbaustoffe, das Zusammenwirken der Teile im Ganzen, das Erzeugen von andern seiner Art. Dieser ganze Prozeß, den wir Leben nennen, ist Tätigsein und Tätigsein ist Bewegung”.

4 Note: the verb grasp is used to avoid a word related to the act of intellectual knowledge, since that actions comes before the thinking, is pre-reflexive.

5 In the Spanish version, *Leib* is translated as living body, but it can also be translated as own body. The latter is used in the Italian version, which we considered is more appropriate. Elio Constantini comments in the Italian version: We have replaced *Leibhaftigkeit* for “own corporality”, which is composed by the noun “*Leib”* (own body) and *Haftigkeit*, which comes from the verb *haften* (to be jointed, attached). The meaning of *Leib* derives from the own body as a living body, a body attached to a vital principle. The term *Leibhaftkei*tis is fundamental for the constitution of the psychophysical individual, but not for that of the personality. Cfr. Contantini, Elio. Introducción y notas a la traducción italiana: Stein Edith, *Il problema dell’empatia.* Ed. Studium, Roma 1985, p. 125–126, note b.

6 The *I* for phenomenology is the “continuous flow of experiences”. For Freud, the *I* is one of the three instances that constitute the psychic apparatus- Id, ego, superego- which is basically ruled by the Principle of Reality and the functioning of secondary processes (perception, thought, language).

7 For this reason, Merleau Ponty states that if phenomenology, according to its interest to reveal the world as it is, has been a movement and not a doctrine, it is not a matter of chance or imposture. This statement echoes that of Paul Ricoeur when he affirms that the history of phenomenology is constituted by the history of Husserlian heresies (*Sur la phénomenologie*, p. 836).
